# Normal Pregnancy-Induced Islet Beta Cell Proliferation in Mouse Models That Are Deficient in Serotonin-Signaling

**DOI:** 10.3390/ijms232415816

**Published:** 2022-12-13

**Authors:** Lotte Goyvaerts, Anica Schraenen, Katleen Lemaire, Peter in’t Veld, Ilse Smolders, Luc Maroteaux, Frans Schuit

**Affiliations:** 1 Gene Expression Unit, Department of Cellular and Molecular Medicine, KU Leuven, 3000 Leuven, Belgium; 2 Department of Pathology, Vrije Universiteit Brussel, 1090 Brussels, Belgium; 3 Department of Pharmaceutical Sciences, Research Group Experimental Pharmacology (EFAR), Center for Neurosciences (C4N), Vrije Universiteit Brussel, 1090 Brussels, Belgium; 4 INSERM UMR-U1270, Institut du Fer à Moulin, Sorbonne Université Paris, 75006 Paris, France

**Keywords:** pregnancy, serotonin, beta cell proliferation

## Abstract

During mouse pregnancy placental lactogens stimulate prolactin receptors on pancreatic islet beta cells to induce expression of the tryptophan hydroxylase *Tph1*, resulting in the synthesis and secretion of serotonin. Presently, the functional relevance of this phenomenon is unclear. One hypothesis is that serotonin-induced activation of 5-HT_2B_ receptors on beta cells stimulates beta cell proliferation during pregnancy. We tested this hypothesis via three different mouse models: (i) total *Tph1*KO mice, (ii) 129P2/OlaHsd mice, which are incompetent to upregulate islet *Tph1* during pregnancy, whereas *Tph1* is normally expressed in the intestine, mammary glands, and placenta, and (iii) *Htr2b*-deficient mice. We observed normal pregnancy-induced levels of beta cell proliferation in total *Tph1*KO mice, 129P2/OlaHsd mice, and in *Htr2b*^−/−^ mice. The three studied mouse models indicate that islet serotonin production and its signaling via 5-HT_2B_ receptors are not required for the wave of beta cell proliferation that occurs during normal mouse pregnancy.

## 1. Introduction

During pregnancy, a subset of beta cells is stimulated by placental lactogens to induce the mRNA levels encoding the non-neuronal isoform of tryptophan hydroxylase *Tph1* [1,2,3,4], an enzyme that catalyzes the rate-limiting step of serotonin biosynthesis [5]. As beta cells constitutively express aromatic amino acid decarboxylase, the upregulation of Tph1 activates the metabolic pathway converting tryptophan into serotonin [1,2,3,4]. 

*Tph1* is responsible for serotonin synthesis outside the brain, which mainly occurs in the intestinal wall, where it influences gut motility. Blood platelets, after having collected intestinal serotonin, regulate vascular tone [6] and proliferation of liver parenchymal cells [7]. Approximately 5% of serotonin is produced as a neurotransmitter in the central nervous system, where it affects various behavioral processes such as mood, appetite, and sexual activity [8]. This production requires the expression of the neuronal isoform of tryptophan hydroxylase, *Tph2*. Curiously, pancreatic islets of pregnant mice also upregulate expression of brain-type *Tph2*, but it is not known whether this event occurs in the hormone secreting cells or in the nerve endings. 

A growing number of studies indicate that serotonin acts at several levels as a regulator of reproductive function, by arousing sexual activity and by supporting normal pregnancy and lactation. Analysis of *Tph1*-deficient mice revealed an interesting genotype/phenotype relationship for fetal development: *Tph1^−/−^* offspring of *Tph1^+/−^* mothers were born normally, while *Tph1^+/−^* offspring of *Tph1^−/−^* mothers developed brain abnormalities [9]. The conclusion of this work was that the production of maternal serotonin is important for normal fetal development [9]. A second observation in the field of reproductive neuroendocrinology is that *Tph1* expression is upregulated in mammary glands during lactation and that serotonin acts as a negative feedback regulator for milk production [10]. Mouse beta cells express high amounts of prolactin receptors [11]. During pregnancy these receptors bind circulating placental lactogens, resulting in the activation of the JAK2/STAT5 signaling pathway, which stimulated beta cell proliferation [1,2]. One of the biological effects is strong upregulation of *Tph1* and activation of serotonin synthesis, which occurs in a subset of the pancreatic beta cell population [1]. The phenomenon of a stimulated serotonin biosynthetic pathway in insulin-secreting cells raised the question, what could be the physiological function of this adaptation [12]? More than ten years later, this question has not been adequately answered. One hypothesis proposes that the serotonin produced in beta cells is responsible for beta cell proliferation during pregnancy [2]. This hypothesis was mainly supported by the observation that beta cell proliferation during pregnancy is diminished in *Htr2b*-deficient mice [2]. However, the often-cited hypothesis still awaits confirmation by other groups. Given the importance to understand beta cell serotonin during pregnancy, and the fact that an often-cited work [2] has not been confirmed by other labs, we further investigated the hypothesis of serotonin/*Htr2b* receptor-regulated beta cell proliferation during pregnancy using three different animal models: (i) *Tph1*KO mice [13], (ii) 129P2/OlaHsd mice, in which the pregnancy-related expression of *Tph1* in islets has been lost, whereas *Tph1* is normally expressed in intestines, mammary glands, and placenta, and (iii) *Htr2b* deficient mice. 

## 2. Results

### 2.1. Tph1KO Mice

#### 2.1.1. Serotonin Biosynthesis in Pancreatic Islets of Total *Tph1*KO Mice

**Figure 1:**
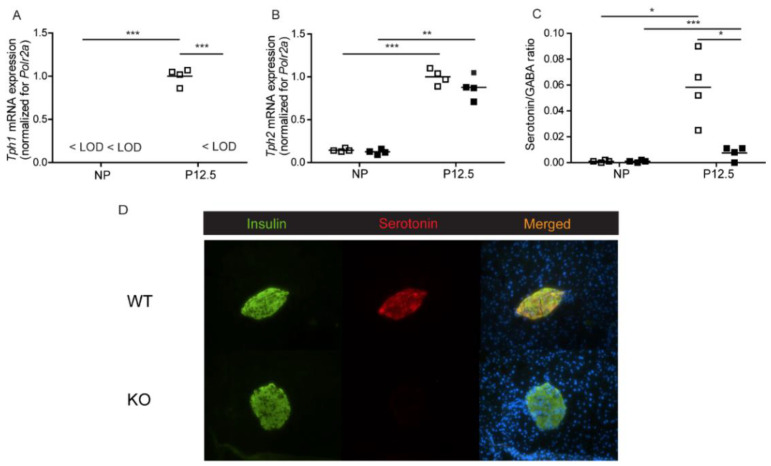
Analysis of serotonin biosynthesis in pancreatic islets of wild-type (□) and total *Tph1*KO mice (■). (**A**): *Tph1* mRNA, quantified by Q-RT-PCR, was below level of detection (<LOD) in islets of non-pregnant animals and in islets of pregnant *Tph1*KO mice; in contrast, *Tph1* mRNA was strongly upregulated in islets of wild-type mice at P12.5. (**B**): Upregulation of *Tph2* mRNA level (quantified by Q-RT-PCR) during pregnancy was comparable in *Tph1*KO mice and wild-type mice. (**C**): Islet serotonin/GABA ratios were significantly lower in pregnant *Tph1*KO mice as compared to pregnant wild-type mice. Each point in (**A**–**C**) represents the measurement of material sampled from one mouse; mean values are represented by horizontal bars. Significance of differences was calculated by unpaired Student’s-*t*-tests: * *p* < 0.05, ** *p* < 0.01, *** *p* < 0.001. (**D**): Absence of serotonin immunoreactivity in pancreatic islets of P12.5 *Tph1*KO mice. Staining for insulin (green), serotonin (red) and DAPI (blue) on pancreatic sections of wild-type and *Tph1*KO mice.

We first assessed pregnancy-induced serotonin biosynthesis in pancreatic islets of total *Tph1*KO mice [13]. Figure 1 shows serotonin levels and tryptophan hydroxylase expression in wild type and knock-out mice. *Tph1* mRNA expression was absent in islets of *Tph1*KO mice and no compensatory mechanism involving *Tph2* was found (Figure 1A,B). Islet serotonin/GABA ratios in the islets were severely diminished in *Tph1*KO mice (Figure 1C); GABA content was not being influenced by the islet genotype. A low level of remaining serotonin remained detectable in the *Tph1*KO islets, which can be attributed to the presence of *Tph2* enzyme. The total loss of *Tph1* expression and the near complete loss of serotonin biosynthesis during pregnancy was also reflected in the disappearance of serotonin immunostaining in a subpopulation of islet beta cells of pregnant *Tph1*KO mice (Figure 1D).

#### 2.1.2. Beta Cell Proliferation in Total *Tph1*KO Mice

**Figure 2:**
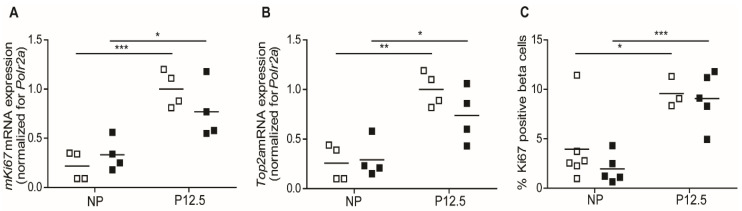
Beta cell proliferation in wild-type (☐) and *Tph1*KO mice (■) in non-pregnant condition and at P12.5. Quantification of *mKi67* (**A**) and *Top2a* (**B**) mRNA using Q-RT-PCR showed comparable pregnancy-induced upregulation in wild-type and *Tph1*KO mice. (**C**): The percentage of Ki67-positive/insulin-positive beta cells, quantified in microscopic pancreatic sections, increased significantly during pregnancy both in wild-type and *Tph1*KO mice. Each point in (**A**–**C**) represents the measurement of material sampled from one mouse; mean values are represented by horizontal bars. Significance of differences between groups were calculated by unpaired Student’s-*t*-tests: * *p* < 0.05, ** *p* < 0.01, *** *p* < 0.001.

In the present study, we used quantitative mRNA expression signals of the cell cycle-related genes, *mKi67* and *Top2a* [14] in pancreatic islet extracts, and nuclear staining of Ki67 of insulin-containing cells as markers for beta cell proliferation. For both *mKi67* (Figure 2A) and *Top2a* (Figure 2B), the upregulation of mRNA signals at 12.5 days of pregnancy was not different in wild-type and *Tph1*KO mice. We next counted, by light microscopy of pancreatic sections, the percentage of proliferating beta cells (double positive for Ki67/insulin immunostaining) and observed no difference between wild type and *Tph1*KO mice both before pregnancy and at P12.5 (Figure 2C). Indeed, we observed a robust (*p* < 0.001) increase in the number of replicating beta cells at P12.5 in *Tph1*KO mice.

#### 2.1.3. Glucose Tolerance in Total *Tph1*KO Mice

**Figure 3:**
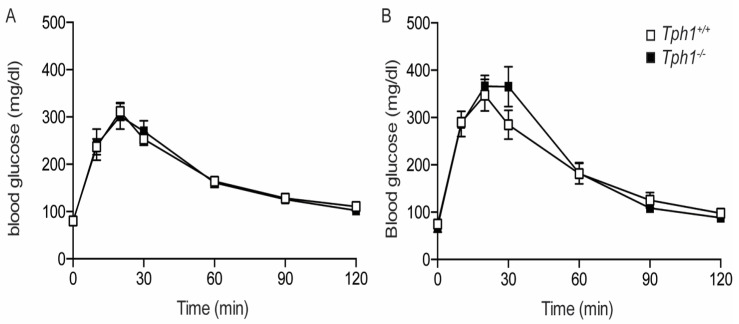
Normal glucose tolerance in NP and P12.5 *Tph1*^−/−^ mice. (**A**): Oral glucose tolerance in NP mice (*Tph1*^+/+^ (☐), *n* = 8 and *Tph1*^−/−^ (■), *n* = 10). (**B**): Oral glucose tolerance in P12.5 mice (*Tph1*^+/+^ (☐), *n* = 4 and *Tph1*^−/−^ (■), *n* = 4).

In *Tph1*KO mice, body weight and random fed blood glucose values did not differ from the control animals (Figure S1). Oral glucose tolerance tests were performed in wild-type and *Tph1*KO mice in NP and P12.5 condition. In non-pregnant animals, *Tph1*^+/+^ and *Tph1*^−/−^ mice were observed to be equally tolerant for an oral glucose bolus (Figure 3A). Also, during pregnancy, oral glucose tolerance in *Tph1*^+/+^ and *Tph1*^−/−^ mice were not significantly different (Figure 3B). Normal glucose tolerance was further documented when comparing the area under the curve values in *Tph1*^+/+^ and *Tph1*^−/−^ mice both in the NP and P17.5 conditions (Figure S2).

### 2.2. 129P2/OlaHsd Mice

#### 2.2.1. Loss of Pregnancy-Induced Regulation *Tph1* in 129P2/OlaHsd Mice

**Figure 4:**
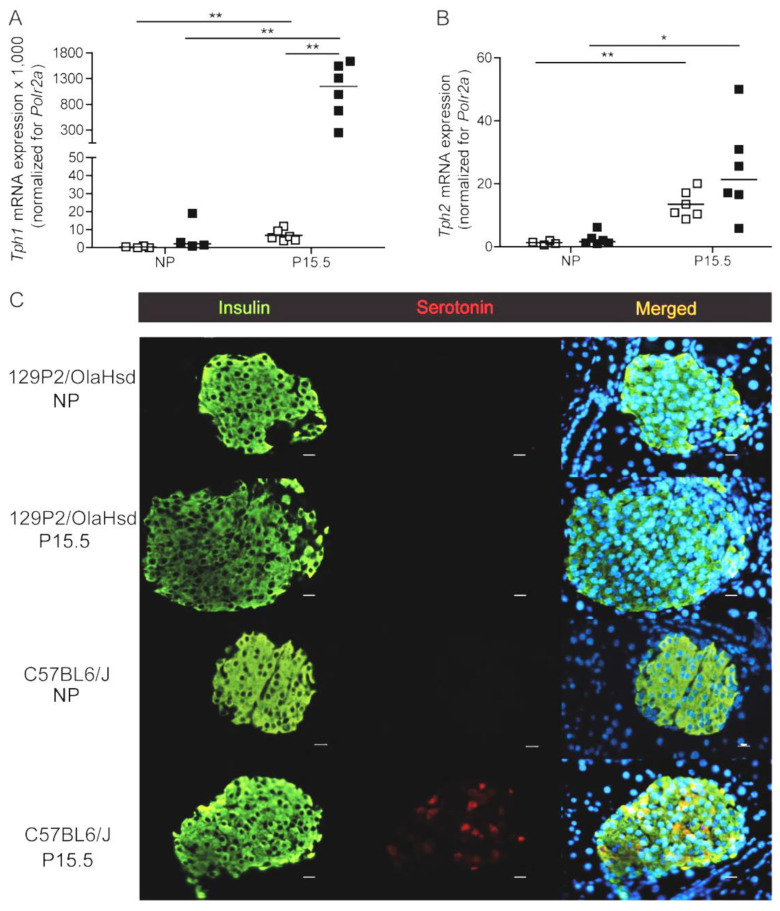
Expression of tryptophan hydroxylase and serotonin immunoreactivity in non-pregnant and pregnant 129P2/OlaHsd versus C57BL6/J mice. Measurement of expression signals of *Tph1* (**A**) and *Tph2* (**B**) in pancreatic islets isolated from 129P2/OlaHsd mice (☐) versus C57Bl6/J mice (■) was performed by Q-RT-PCR. Each point in (**A**,**B**) represents the measurement of material sampled from one mouse; mean values are represented by horizontal bars. Data were normalized for the housekeeping gene *Polr2a* and the signal ratio in islet from non-pregnant 129P2/OlaHsd mice was set at 1.0. Significance of differences between groups was calculated using the unpaired Student’s-*t*-tests * *p* < 0.05, ** *p* < 0.01. (**C**): Absence of detectable islet serotonin immunoreactivity in pancreatic sections of 15.5 days pregnant 129P2/OlaHsd mice. Immunostaining for insulin (green) and serotonin (red) was performed on pancreatic sections of non-pregnant and P15.5 pregnant 129P2/OlaHsd compared with C57BL6/J mice. Nuclei are stained with DAPI (blue). Magnification 40×, scale bars 10 µm.

Body weight of the 129P2/OlaHsd and C57BL6/J strains was not significantly different (Figure S3A). Blood glucose was, however, significantly higher in C57BL6/J mice (Figure S3B). In contrast to the strong upregulation of *Tph1* and *Tph2* mRNA during pregnancy in pancreatic islets of C57BL6/J mice (Figure 4A,B), we observed no effect of pregnancy on *Tph1* mRNA in islets isolated from 129P2/OlaHsd mice (Figure 4A,B), while *Tph2* mRNA was upregulated like we measured in islets from C57BL6/J mice. The loss of pregnancy-induced upregulation *Tph1* in 129P2/OlaHsd mice was associated with an absence of detectable serotonin immunostaining of pancreatic islets of P15.5 pregnant 129P2/OlaHsd mice (Figure 4C), such as occurs in the total *Tph1*KO mouse model (Figure 1D).

#### 2.2.2. *Tph1* Expression in Intestine, Placenta, and Mammary Glands from 129P2/OlaHsd Mice

**Figure 5:**
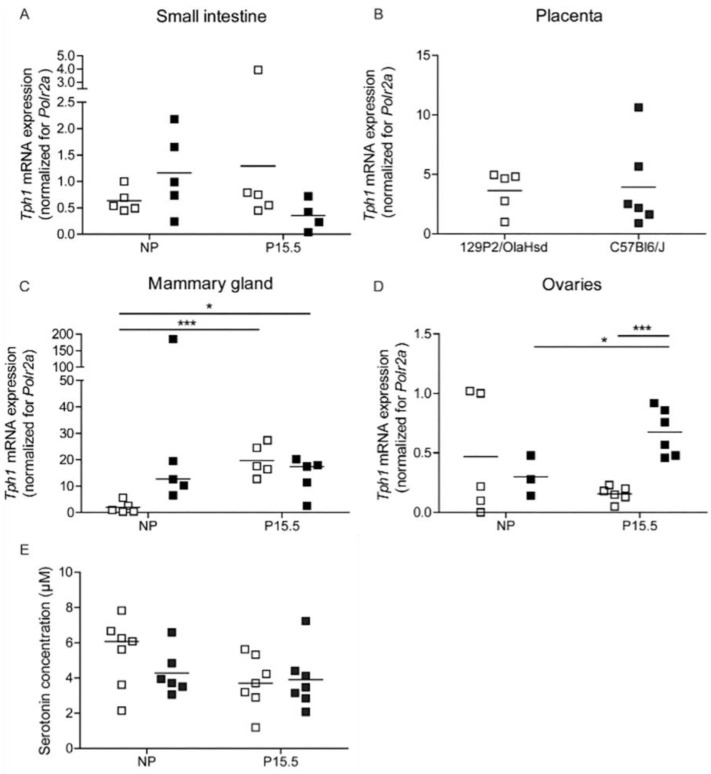
Regulation of *Tph1* expression in 129P2/OlaHsd (☐) and C57BL6/J mice (■). Each point in (**A**–**D**) represents the Q-RT-PCR measurement of material sampled from one mouse; mean values are represented by horizontal bars. Significance of differences between groups was calculated using Student’s-*t*-tests. * *p* < 0.05, *** *p* < 0.001. *Tph1* mRNA expression was not significantly different in the two mouse strains in small intestine (**A**), placenta (**B**), and mammary glands (**C**). However, in ovaries, Tph1 levels are more upregulated in C57Bl6/J mice as compared to 129P2/OlaHsd mice (**D**). Data were normalized for the Polr2a and expressed relative mRNA level of the non-pregnant 129P2/OlaHsd group (**E**): Blood serotonin levels are similar in all conditions.

The loss of pregnancy-induced regulation *Tph1* in 129P2/OlaHsd mice was accompanied by more subtle abnormalities of *Tph1* expression in other tissues (Figure 5). In extracts of small intestine, no significant difference between C57BL6/J and 129P2/OlaHsd mice could be detected (Figure 5A). Also, in placenta, *Tph1* mRNA signals were comparable in both mouse strains (Figure 5B). However, in mammary glands, *Tph1* mRNA was more upregulated by pregnancy in 129P2/OlaHsd mice than in C57BL6/J mice, which had higher expression signals in the non-pregnant condition (Figure 5C). In ovaries, a comparable result was found as we observed, in islets, a pregnancy-induced increase in *Tph1* expression in the C57BL6/J mice, which was not detected in 129P2/OlaHsd mice (Figure 5D). These subtle differences in *Tph1* expression in different organs had no repercussion on blood serotonin levels, which were comparable in the different groups (Figure 5E). 

#### 2.2.3. Beta Cell Proliferation in Pregnant 129P2/OlaHsd Mice

**Figure 6:**
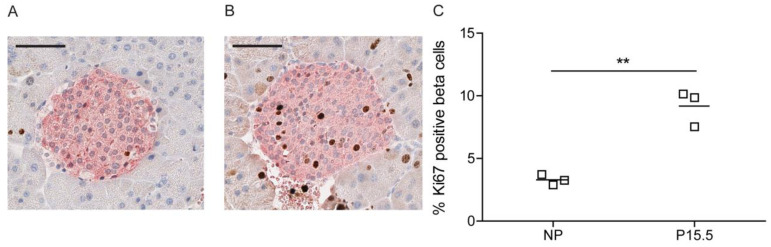
Pregnancy induces a normal level of beta cell proliferation in 129P2/OlaHsd mice. Representative immunohistochemical staining of a pancreatic islet of a NP (**A**) and a P15.5 (**B**) 129P2/OlaHsd mouse. The nuclei of the pancreatic cells were stained with haematoxylin (blue) and for the proliferation marker Ki67 (brown). Insulin-positive beta cells are labeled pink. Scale bar 50 µm. (**C**): Quantification of beta cell proliferation (% Ki67 positive beta cells) in NP and P15.5 129P2/OlaHsd mice. Significance of differences between non-pregnant and pregnant mice was calculated using the unpaired Student’s-*t*-tests ** *p* < 0.01, *n* = 3 for each group.

We next tested the hypothesis in which serotonin is responsible for the stimulation of beta cell proliferation during pregnancy. Using nuclear staining of Ki67 of insulin-containing cells as a marker for beta cell proliferation, we observed a three-fold increase in beta cell proliferation when comparing non-pregnant and P15.5 pregnant 129P2/OlaHsd mice (Figure 6); this upregulation was comparable to the data observed in wild type C57Bl6/J mice (Figure 2C).

### 2.3. Htr2b Deficient Mice

#### 2.3.1. Low Expression Levels of *Htr2b* in Mouse Islets as Compared to Placenta

**Figure 7:**
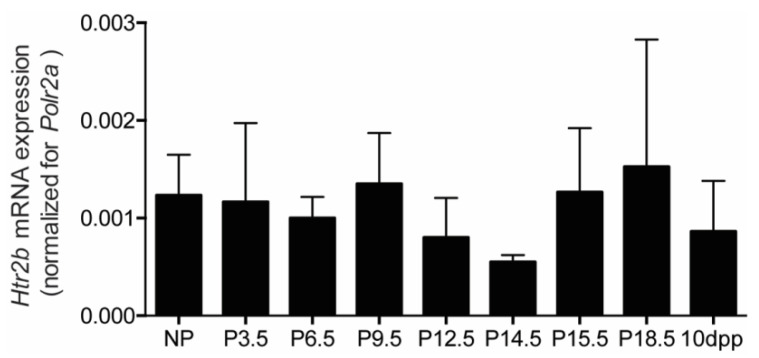
Pancreatic islet *Htr2b* mRNA expression signals during pregnancy and lactation. mRNA was extracted from isolated islets at different time points of pregnancy and 10 days postpartum (*n* = 3 to 9 independent islet preparations for each time point). *Htr2b* mRNA was normalized for *Polr2a* expression in the same tissue sample; islet expression levels were expressed as a ratio of the measured placental mRNA signal.

The third mouse model (*Htr2b* knockout mice) in which we tested the hypothesis of serotonin-induced beta cell proliferation departed from the idea that 5-HT_2B_ receptors on beta cells mediate the mitogenic effect of serotonin [2]. When analysing a large set of mouse tissues in wild type mice, we observed that *Htr2b* mRNA signals were highest in the placenta and lowest in pancreatic islets. Indeed, as can be seen in Figure 7, the *Htr2b* mRNA expression level in islets is very low, approximately 1000 times less than the signal measured in placenta (Figure 7). We did not observe any significant change of the low expression level of *Htr2b* mRNA during pregnancy or lactation (Figure 7). 

#### 2.3.2. Expression of *Htr2b* mRNA, *Tph1*, and *Tph2* mRNA in *Htr2b* Deficient Mice

**Figure 8:**
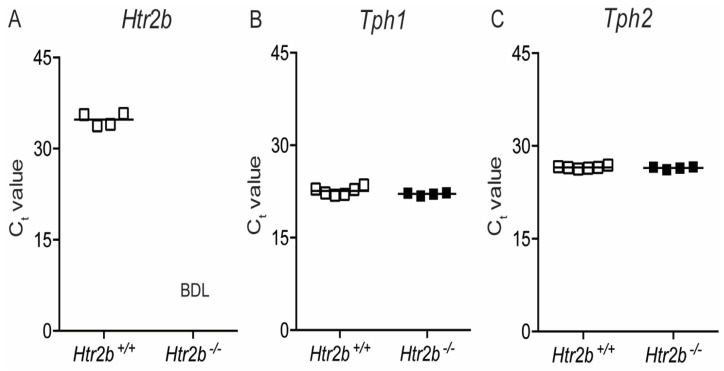
Expression of *Htr2b*, *Tph1*, and *Tph2* mRNA in pancreatic islets of *Htr2b*^+/+^ (☐) and Htr2b^−/−^ mice (■) at P18.5 of pregnancy. Data of qRT-PCR analysis of *Htr2b* (**A**), *Tph1* (**B**) and *Tph2* (**C**) are shown, each square representing the analysis of islets of one mouse, and—representing the mean C_t_ value. BDL = Below the Detection Limit. *Polr2a* C_t_ values from the same islet extracts are shown in Figure S4.

Figure 8A shows that the low expression signal of *Htr2b* mRNA in *Htr2b*^+/+^ mice compares with undetectable *Htr2b* mRNA expression in *Htr2b*^−/−^ mice. This loss of receptor expression has no influence on the expression of tryptophan hydroxylases, shown in Figure 8B,C as C_t_ values observed in extracts from islets isolated at P18.5 of pregnancy.

**Figure 9:**
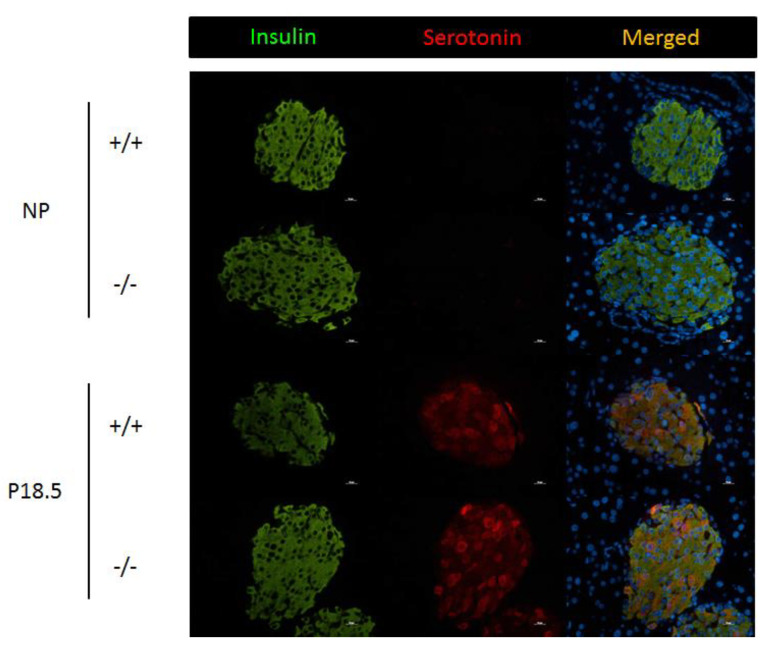
Serotonin immunoreactivity in pancreatic islets from non-pregnant (NP) and pregnant (P18.5) wild type (+/+) and *Htr2b*^−/−^ mice (−/−). Pancreatic sections were stained for insulin (green), serotonin (red), and DAPI (blue). Scale bars 10 µm.

The presence of immunoreactive serotonin was next assessed in pancreatic sections of *Htr2b*^+/+^ and *Htr2b*^−/−^ mice, both before pregnancy and in 18.5 days pregnant females. As was expected from previous experiments, no serotonin-positive beta cells could be detected in pancreatic sections from non-pregnant females (Figure 9). Furthermore, we observed heterogenous immunoreactivity serotonin (staining a subpopulation of beta cells) in pancreata from pregnant females; no differences between pancreata from *Htr2b*^+/+^ and *Htr2b*^−/−^ mice could be found (Figure 9).

#### 2.3.3. Beta Cell Proliferation in *Htr2b* Deficient Mice

**Figure 10:**
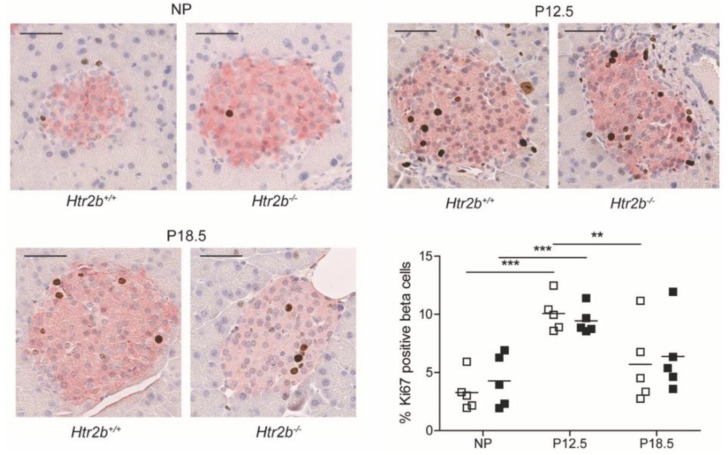
Normal pregnancy-stimulated beta cell proliferation in *Htr2b*^−/−^ mice. Representative images show one pancreatic islet from *Htr2b*^+/+^ and *Htr2b*^−/−^ mice that were studied in non-pregnant animals or during pregnancy (P12.5 and P18.5). Proliferating insulin-positive beta cells with nuclear presence of Ki67 (pink cytoplasm and brown nucleus) were counted. The highest levels of beta cell proliferation were P12.5 in both *Htr2b*^+/+^ mice (☐) and in *Htr2b*^−/−^ mice (■). Each square represents the analysis of one mouse pancreas, and—represents the mean. ** *p* < 0.01, *** *p* < 0.001.

As in the other two mouse models, beta cell proliferation rates were estimated using the immunohistochemical markers of nuclear Ki67 staining and cytoplasmic insulin staining. In this study we measured Ki67/insulin positive cells at three time points in both genotypes: NP, P12.5, and P18.5. As we published before for wild type animals [14], a peak of the beta cell proliferation rate was observed at P12.5 in both strains (Figure 10). As expected, proliferation rates had declined again to near basal levels at P18.5. In none of the studied conditions, a difference between *Htr2b*^+/+^ and *Htr2b*^−/−^ mice could be observed (Figure 10).

#### 2.3.4. Glucose Tolerance in *Htr2b* Deficient Mice

**Figure 11:**
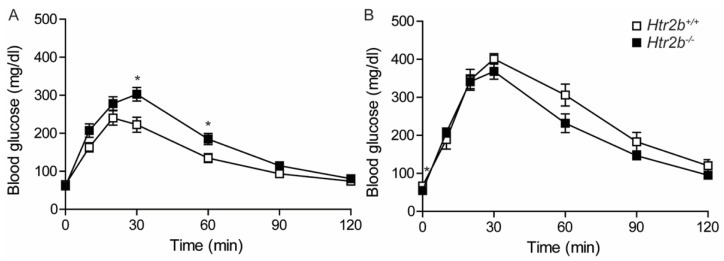
Oral glucose tolerance in *Htr2b*^+/+^ (☐) and in *Htr2b*^−/−^ (■) mice. (**A**): Oral glucose tolerance of non-pregnant females (*n* = 10 for Htr2b^+/+^ and *n* = 12 for *Htr2b*^−/−^). (**B**): Oral glucose tolerance of P17.5 pregnant *Htr2b*^+/+^ (☐ *n* = 4) and *Htr2b*^−/−^ mice (■ *n* = 10). Data are presented as mean ± SEM. * *p* < 0.05.

Glucose tolerance of wild-type and Htr2b deficient mice was measured both in non-pregnant females (Figure 11A) and during pregnancy (P17.5, Figure 11B). Differences between the two strains were small and not consistent. Indeed, in non-pregnant animals, Htr2b^−/−^ mice were slightly less glucose tolerant than wild type mice. However, during pregnancy, Htr2b^−/−^ mice had slightly lower blood glucose levels 30–120 min after stimulation. Body weight and random fed blood glucose levels were also not different when comparing of wild-type and Htr2b deficient animals (Figure S5).

## 3. Discussion

In 2010, two laboratories reported that serotonin biosynthesis is activated in insulin-secreting cells of pregnant mice, the mechanism being strong placental lactogen-induced upregulation of expression of the non-neuronal isoform of tryptophan hydroxylase [1,2]. Kim et al. proposed an autocrine/paracrine signaling pathway in which the secreted serotonin activates 5-HT_2B_ receptors on beta cells [2]. One of the main observation was that beta cell proliferation during pregnancy was not present in *Htr2b* deficient mice [2]. The loss of proliferation was considered to be physiologically relevant, as the pregnant *Htr2b^−/−^* mice became glucose intolerant [2]. The idea that serotonin can stimulate beta cell proliferation seems to match with an earlier observation that liver parenchymal cell regeneration is stimulated by blood platelet-derived serotonin [7]. 

Beta cell proliferation is an important topic of diabetes research, and perhaps crucial in order to understand the mechanism by which gestational diabetes develops. Of interest is that more than 10 years after publication, the serotonin-induced beta cell proliferation hypothesis [2] has not been challenged nor corroborated by other laboratories. We used, in the present study, three different mouse models, including the model used by Kim et al. [2] to test whether or not serotonin production in mouse islet beta cells is relevant for the extensively studied wave of beta cell proliferation that peaks around mid-pregnancy [14,15,16]. In these models, we quantified on the one hand, mRNA expression signals of the cell cycle-related genes *mKi67* and *Top2a* [14] in pancreatic islet extracts as markers for islet cell proliferation. On the other hand, we quantified proliferating beta cells in pancreatic sections by counting insulin-containing cells that exhibited nuclear staining of Ki67.

The first model to be discussed is the *Tph1* knockout mouse in which tryptophan hydroxylase 1-mediated serotonin biosynthesis is completely inactivated in homozygous animals with an inactivating mutation of the gene [13]. Interestingly, the female homozygous mouse has a pregnancy-related phenotype: the offspring of *Tph1*^−/−^ mothers are born with cardiac and central nervous system abnormalities [13], while homozygous null mutant fetuses develop normally when growing in heterozygous mothers [13]. 

In the present study, we show that expression of tryptophan hydroxylase 1 is undetectably low in islets isolated from pregnant mice, and islet serotonin is undetectably low, both in biochemical assays and using light microscopic immunostaining. This loss did not result in glucose intolerance. Moreover, using two techniques to measure beta cell proliferation, we observed a normal response in pregnant *Tph1*^−/−^ mice, with peak levels of proliferating beta cells at P12.5. The first method was quantitative RT-PCR measuring, in islet extracts, the expression of mRNA encoding cell cycle-related genes *mKi67* and *Top2a* [14]. The second method consisted of light microscopic identification of proliferating islet beta cells in pancreatic sections from pregnant and non-pregnant mice, in which positive cells were defined by both cytoplasmic insulin staining and nuclear Ki67 immunostaining. We repeated these experiments sufficiently to conclude that there was no statistically relevant difference between wild type and *Tph1*^−/−^ mice of beta cell proliferation, while *Tph1*^−/−^ mice are severely restricted in their capacity to produce serotonin during pregnancy. One concern is that in this model, the paralogous *Tph2* gene is not inactivated, so that the inactivation of *Tph1* could drive compensatory ectopic expression or hyperexpression of *Tph2*. We did, however, observe *Tph2* expression signals in islets from *Tph1KO* mice that were indistinguishable from signals in wild type animals and we conclude that there is no compensatory response of the *Tph2* gene in the *Tph1*^−/−^ mice. It should be repeated here that it is presently unknown which cell type in the islet (endocrine, neural, duct cells) is responsible for the RT-PCR mRNA signal *Tph2*. Furthermore, compared to *Tph1*, *Tph2* enzymatic activity is likely to be very low, as no immunoreactive serotonin was found in islets from *Tph1*^−/−^ mice. Another concern about the whole-body *Tph1*^−/−^ mice is the loss of serotonin in one or several tissues that may have altered regulatory signaling loops in a (neuro)endocrine network. This might complicate the relationship between beta cell serotonin and beta cell proliferation by unknown confounders. 

This concern brings us to the second model in this study. When analyzing the phenotype of prolactin receptor-deficient mice [17], we observed that the genetic background of heterozygous *Prlr*^+/−^ mice (129P2/OlaHsd versus C57Bl6/J) was correlated with strain-specific differences in *Tph1* mRNA expression levels. These differences seemed to be of interest, as an earlier study described that *Prlr^+/−C57Bl6/J^* females could not lactate, while *Prlr^+/−129 P2/OlaHsd^* females could lactate from their second litter [18]. *Tph1*-dependent serotonin production in the mammary glands has been shown to be an important feedback inhibitor for lactation [19]. Therefore, we were interested to search for possible strain-specific traits at the level of *Tph1* expression and serotonin production and, therefore, we examined mammary glands, placenta, ovaries, small intestine, and pancreatic islets of female 129P2/OlaHsd versus C57BL6/J mice (pregnant and non-pregnant). As we presented in Figure 4 and Figure 5, a major difference of serotonin production is present in pancreatic islets. In the 129P2/OlaHsd-genetic background, we observed no pregnancy-induced induction of *Tph1* mRNA and serotonin immunoreactivity in islets isolated from P15.5 pregnant mice was undetectably low. In contrast, in other tissues we measured small differences in the level of *Tph1* mRNA expression levels when comparing 129P2/OlaHsd and C57BL6/J mice and blood serotonin levels were comparable in the two strains. In agreement with the abovementioned results in whole-body *Tph1*^−/−^ mice, significant levels of stimulation of beta cell proliferation were found during pregnancy in 129P2/OlaHsd mice. Together, in a spontaneous mouse model of islet selective unresponsiveness of the *Tph1* gene to pregnancy, we reached the same conclusion as in the whole-body *Tph1*^−/−^ mice: the presence of serotonin in pancreatic beta cells is not required for a normal level of beta cell proliferation during pregnancy. At this moment we do not know via what mechanism regulation of the *Tph1* gene is altered in the 129P2/OlaHsd background. We have sequenced the STAT5 binding site of the *Tph1* promoter [20] in both strains but found no mutation in this region in the *Prlr^+/+129 P2/OlaHsd^* mice. In the literature, other phenotypic differences between the C57BL6/J and 129P2/OlaHsd strains, such as the response of liver tissue to high fat diet [21], have been described. As the offspring of 129P2/OlaHsd mice develop normally (no brain and heart phenotype has been described), it is unlikely that the source of maternal serotonin that is needed for normal fetal development is endocrine secretion from pancreatic beta cells (see discussion of the work of Cote et al. [13] above). This is not the first spontaneous mutation of the serotonin metabolic pathway; a well-known example of a natural knockout mouse is the C57Bl6/J strain, which has a mutation in the gene encoding the enzyme serotonin N-acetyltransferase, so that this strain is not competent to produce melatonin [22].

In the present study we also tested the relevance of serotonin 2B receptors for beta cell proliferation, and we used the same genetic model, *Htr2b* deficient mice [19], as was used by Kim et al. [2]. The first point to be discussed is the weakness of the *Htr2b* mRNA-expression signal in mouse pancreatic islets. In our hands, it was only possible to detect a qRT-PCR signal when the starting amount of cDNA was increased 10 times over the standard amount used for other genes. The low mRNA expression signal in pancreatic islets was a contrast with the approximately one thousand-fold stronger signal we measured in placenta (the reference tissue in our study). Second, when we compared the *Htr2b* mRNA-expression signal in islets from non-pregnant and pregnant mice, we were unable to observe an effect of pregnancy on gene expression. Our result is different from what was published by Kim et al. [2] who reported upregulation of *Htr2b* mRNA-expression during pregnancy. We have no explanation for this discrepancy. Despite the low *Htr2b* mRNA-expression signals in islets, we could demonstrate complete loss of expression in pancreatic islets from *Htr2b* KO mice and observed that pregnancy-induced beta cell proliferation in this model is normal. In contrast, Kim et al. [2] reported a loss of pregnancy-induced beta cell proliferation in *Htr2b* KO mice. Differences exist in the chosen methods in the two studies. In our own study we counted insulin-positive beta cells with nuclear Ki67 staining in islets present in pancreatic sections. Kim et al. analyzed *Htr2b^+/+^* and *Htr2b^−/−^* islets that were transplanted under the kidney capsule of *Htr2b^+/+^* mice using BrdU staining. Prior to transplantation, the endogenous beta cells of the acceptor mice were not chemically destroyed [2]. Finally, both studies differ in the reported metabolic phenotype; while Kim et al. [2] reported glucose intolerance *Htr2b^−/−^* mice during pregnancy, we observe that glucose tolerance of *Htr2b^−/−^* mice during pregnancy is normal. On the contrary, we observe minor abnormalities at 30 and 60 min after glucose injection in non-pregnant mice. One explanation for the latter is that glucose is absorbed more rapidly in the *Htr2b^−/−^* mice because 5-HT_2B_ receptors are highly expressed in the gastrointestinal system [23]. 

Why are our results so different from the earlier study by Kim et al.? Some differences in the methods used may play a role. In our study, we used Ki67 protein expression as a marker for beta cell proliferation. In contrast, Kim et al. transplanted *Htr2b^+/+^* and *Htr2b^−/−^* islets under the kidney capsule of *Htr2b^+/+^* mice; prior to transplantation, beta cells of the acceptor mouse were not destroyed by streptozotocin. BrdU staining was then performed on the graft and the pancreas. Another difference between the two studies is the background of the *Htr2b* deficient mice. In our study, the mice were on the 129S2/SvPas background versus C57Bl6/J background in the study by Kim et al. Since the latter are known to be more prone to develop glucose intolerance [24], this could partly explain the differences in the glucose tolerance tests. A third difference is the way glucose tolerance tests were performed: we used oral glucose tolerance tests, while Kim et al. used intraperitoneal glucose tolerance tests. Another difference is the day of pregnancy chosen for experiments: Kim et al. used day 13 of pregnancy. However, because the beta cell proliferation peak is only at P12.5-P15.5 [4,14] and because the peak of serotonin production is at P15.5 [1], we chose P17.5 to perform the oral glucose tolerance tests.

We know from our previous analysis of mRNA expression signals in islets from pregnant mice, published as a twin paper [1,14], that beta cell proliferation can be quantified by the concerted upregulation of expression of a very large amount of cell cycle genes comprising *mKi67* and *Top2a* [14]. In the same analysis [1] we confirmed earlier observations [15,16] that the initiation of these events is triggered by placental lactogens, which bind with high affinity to prolactin receptors on beta cells. We have subsequently shown that the same events can be triggered in male beta cells when transplanted into a female donor mouse [17]. In these studies, the strong upregulation of a serotonin biosynthetic pathway was another remarkable phenotypic trait. But our present work demonstrates that beta cell serotonin is not required for beta cell proliferation. 

A final point of discussion is the heterogenous nature of serotonin production in pancreatic islets, which occurs in a subpopulation of beta cells of pregnant mice. Phenotypic heterogeneity is a hallmark of the glucose-responsiveness of insulin synthesis and insulin secretion of rodent beta cells [25,26] and this phenomenon has been proposed to depend on intercellular differences in glucose metabolism [27]. As the physiological role for beta cell serotonin is still unclear, the relevance for the outspoken intercellular differences in serotonin content observed after immunostaining for insulin and serotonin is unknown. It is conceivable that all beta cells undergo a phasic shift between synthesis and release of the neurotransmitter in which the individual beta cells are not synchronized. Alternatively, the *Tph1* gene is not activated in all beta cells because prolactin receptors are not homogeneously expressed in the beta cell population, or because the JAK2/STAT5 signaling is not equal in individual beta cells. Alternatively, prolactin receptor activation synergizes with other factors (still unidentified) that act on certain beta cells only. One possibility is the anatomic position of beta cells in the pancreatic islet. However, the contribution of a co-signal from sympathetic or parasympathetic nerve endings is unlikely, as we have observed comparable heterogeneity of serotonin immunoreactivity in sections of male islets that were transplanted under the kidney capsule of pregnant female acceptor mice [23], and we could also detect heterogenous of serotonin production in mouse insulinoma cells cultured in the presence of placental lactogen [1].

Together, our analysis of three different mouse models demonstrates that the serotonin content and presence of 5-HT_2B_ receptors on beta cells is irrelevant for the level of beta cell proliferation during pregnancy. 

## 4. Materials and Methods

### 4.1. Animals

#### 4.1.1. Mouse Models

Total *Tph1*KO mice were donated by the lab of Dr. Vodjdani (INSERM, Paris) [13] and the serotonin 2B receptor KO mice originate from the lab of Dr. Maroteaux (INSERM, Paris, France) [19]. All experiments on these animals were approved by the Ethical Committee for Laboratory Animals (ECD) of the KU Leuven (permit numbers: P124/2012, P198/2015) following the guidelines for animal welfare and experimental conduct.

#### 4.1.2. Oral Glucose Tolerance Tests

Mice for oral glucose tolerance tests (OGTT) were fasted overnight (±16 h). In the morning, fasted blood glucose was measured using AccuCheck glucostrips. A glucose bolus (2.5 mg/g body weight) was administered through an oral gavage. Blood glucose was measured at different time points (0, 10, 20, 30, 60, 90, and 120 min).

#### 4.1.3. Isolation of Mouse Pancreatic Islets

Mice were anaesthetized with an intraperitoneal injection of Nembutal (0.01 mL/mg BW) (Ceva, Brussels, Belgium). Immediately after decapitation, a catheter was introduced in the pancreatic duct to inject collagenase P (Roche) in the pancreas. The pancreas was digested at 37 °C in a shaking water bath for 3 min followed by dispersion by pipetting. Pancreatic islets were handpicked in HEPES Krebs buffer (20 mM HEPES, pH 7.4, 119 mM NaCl, 4.75 mM KCl, 2.54 mM CaCl_2_, 1.2 mM MgSO_4_, 1.18 mM KH_2_PO_4_, and 5 mM NaHCO_3_) with 5 mM glucose and 0.5% BSA using a cold light source and a dissecting microscope.

### 4.2. Pancreatic Islet Immunohistochemistry

Pancreata were fixed for 24–48 h in 4% phosphate buffered formaldehyde and embedded in paraffin. We stained 5 µm sections with double immunofluorescence for the detection of serotonin and with double immunohistochemistry for the detection of beta cell replication. For the detection of serotonin, sections were incubated with guinea-pig anti-insulin (a gift from Dr. C. Van Schravendijk, Vrije Universiteit Brussel) and rabbit anti-serotonin (Immunostar, Hudson, WI, USA). Primary antibodies were detected using fluorescein labeled anti-guinea-pig IgG and Cy3 labeled anti-rabbit IgG (both from Jackson Immunoresearch laboratories, West Grove, PA, USA). Sections were mounted with fluorescence mounting medium (Agilent Technologies, Heverlee, Belgium) containing DAPI (10 µg/mL) as a nuclear stain. For the detection of replicating beta cells, sections were incubated with guinea pig anti-rat insulin (gift from Dr. C. Van Schravendijk, Vrije Universiteit Brussel) and rabbit anti-Ki67 (clone SP6, Acris Antibodies, Herford, Germany). Binding of the primary antibody was detected using biotinylated anti-guinea pig and anti-rabbit IgG (Vector Laboratories, Burlingame, CA, USA) in combination with Vectastain Elite ABC kit and Vectastain ABC-AP kit (Vector Laboratories) using diaminobenzidine and Fuchsin as chromogen (Agilent Technologies). Sections were counterstained with haematoxylin. Insulin-Ki67 double positive cells were counted in 1500 insulin positive cells per animal at 400-fold magnification. 

### 4.3. Serotonin and GABA Content

Serotonin was measured in maternal blood. Blood samples for serotonin measurement were taken using heparin as an anticoagulant. Full blood samples were diluted 1:10 in antioxidant (0.1 N perchloric acid, 0.05% Na_2_EDTA, 0.05% Na-metabisulphite) and stored at −80 °C. Serotonin content of samples was measured using a sensitive microbore liquid chromatography method as described in [28]. Islet serotonin content was normalized for the number of islet beta cells by simultaneously measuring GABA, an amino acid that is present in rodent beta cells [29]. GABA content of the islet samples was determined using microbore liquid chromatography as described in [30].

### 4.4. RNA Extraction

RNA was extracted from mouse tissues via two different approaches. RNA of islets of Langerhans was isolated using a kit (Absolutely RNA micro/nanoprep, Agilent, Waldbronn, Germany) according to the manufacturer’s guidelines. TRIzol (ThermoFisher scientific, Waltham, MA, USA) was used for the RNA of other tissues. A spectrophotometer (ND-1000, NanoDrop Technologies, Wilmington, DE, USA) was used to determine the quality of the RNA. 

### 4.5. Quantitative RT-PCR

Total RNA was converted into cDNA using the RevertAid H Minus First Strand cDNA Synthesis Kit (ThermoFisher Scientific, Waltham, MA, USA). Quantitative Real-Time PCR was performed on the cDNA with a Rotorgene (Corbett Research, Mortlake, NSW, Australia) to analyze gene expression levels of different genes in different tissues. An overview of the used primers and Taqman probes (Sigma Aldrich Chemie, GmbH, Deisenhofen, Germany) and Taqman assays (Applied Biosystems) can be found in Table 1. The relative expression levels were calculated with the Pfaffl method [31]. The gene encoding RNA polymerase II (*Polr2a*) was used for normalization of the data.

### 4.6. Statistics

All data are presented as mean ± SEM. Statistical significance was analyzed using a two-tailed Student’s t-test or Welch’s t test, depending on the variance of the samples. 

## 5. Conclusions

We studied beta cell proliferation during pregnancy in three mouse models: whole body *Tph1*-deficient mice, islet-specific deficiency of *Tph1* in 129P2/OlaHsd mice, and *Htr2b*-deficient mice. We observed normal rates of beta cell proliferation during pregnancy in these models. We conclude that the hypothesis of serotonin-induced, 5-HT_2B_ receptor-dependent beta cell proliferation during pregnancy [2] is not supported by our data.

## Figures and Tables

**Table 1:** Primers and probes used for quantitative RT-PCR.

Gene	Primer/Probe	Sequence
*Tph1*	Forward	5′-ATGCACAGCACCACATTTA-3′
	Reverse	5′-CAGTACGGTAAACTCACATGA-3′
	Probe	5′-(6FAM)TCAACTGTTCTCGGCTGATGTCGCAGTCA(TAMRA)-3′
*Tph2*	Taqman assay	Mm00557717_m1 (Applied Biosystems)
*Htr2b*	Forward	5′-TGCATTCATCAAGATTACAGTGGTA-3′
	Reverse	5′-TCAGCTCACAGGTGACATTG-3′
	Probe	5′-(6-FAM)CAATAGGCATCGCCATCCCAGTCCCTAT(TAMRA)-3′
*Polr2a*	Forward	5′-GCACCACGTCCAATGATATTGTG-3′
	Reverse	5′-GGAGATGACATGGTACAGTTCTCG-3′
	Probe	5′-(6-FAM)CTTCCGCACAGCCTCAATGCCCAGT(TAMRA)-3′
*mKi67*	Forward	5′-AGTTCTACCAATCCAACTC-3′
	Reverse	5′-TTGGCTTGCTTCCATCCT-3′
	Probe	5′-(6-FAM)AGACATAATAACCATCATTGACCGCTCCTT(TAMRA)-3′

## Data Availability

Not applicable.

## Supplementary Materials

The following supporting information can be downloaded at: https://www.mdpi.com/article/10.3390/ijms232415816/s1.
