# Supplementary material for: Normal Pregnancy-Induced Islet Beta Cell Proliferation in Mouse Models That Are Deficient in Serotonin-Signaling
Source: Int J Mol Sci. 2022 Dec 13;23(24):15816. doi: 10.3390/ijms232415816 (PMC9779327; doi:10.3390/ijms232415816)
Supplement: Supplementary file 1 [file ijms-23-15816-s001.zip › ijms-2074073-supplementary.pdf]

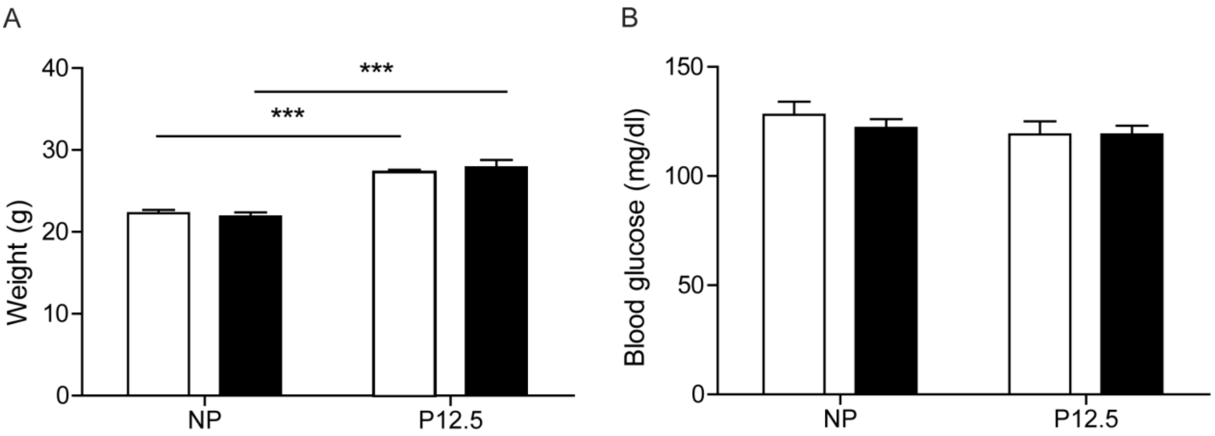

***Figure S1 Body weight and random fed blood glucose values in wild type (□) and total Tph1KO (■) mice. A:** Body weight was significantly higher at P12.5 in both groups. B: Blood glucose values did not differ between both groups. Data are presented as mean ± SEM. *p<0.05, **p<0.01, ***p<0.001.*

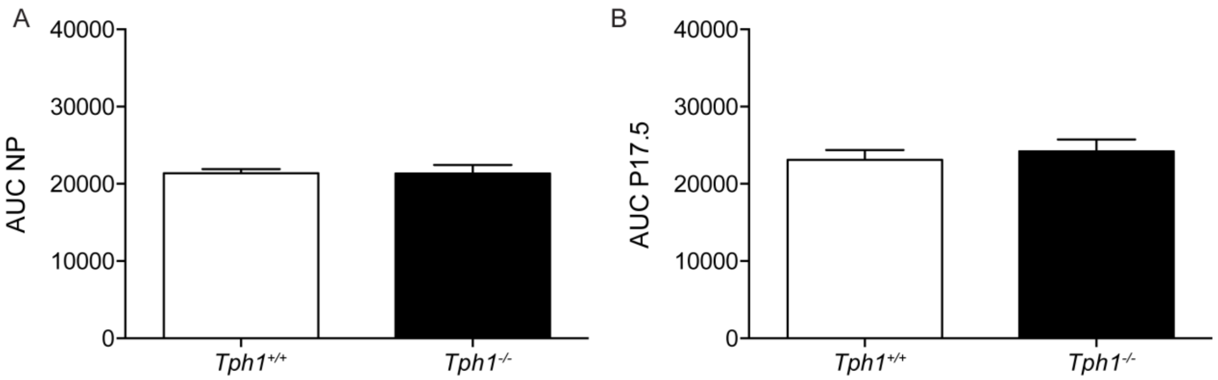

Figure S2 Area under the curve of OGTTs of Tph1 $^{+/+}$ ***(□) and Tph*** $^{-/-}$ ***(■) mice. A:** AUC of OGTT of NP mice showed no difference between groups.* ***B:*** *AUC of OGTT of mice at P17.5 were indifferent between groups. Data are presented as mean ± SEM. *p<0.05.*

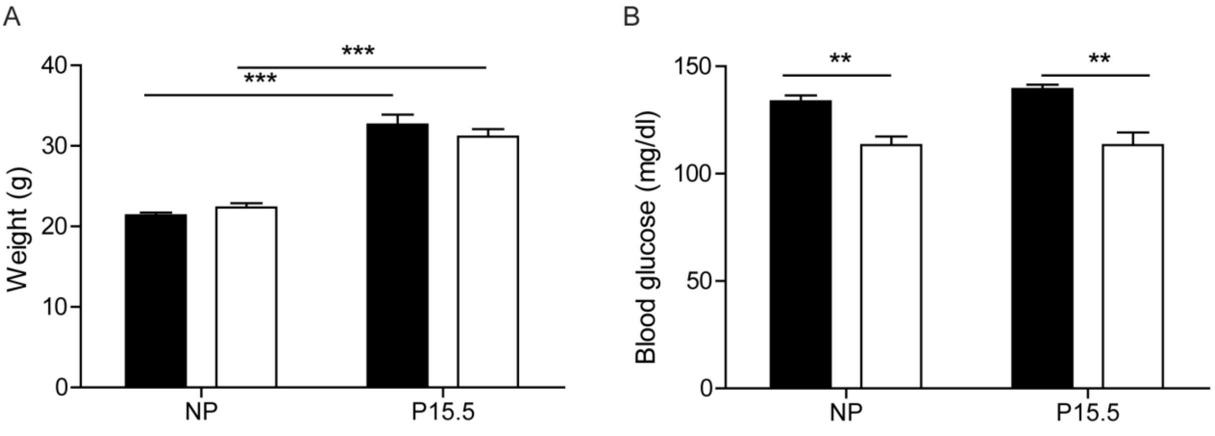

Figure S3 Body weight and random fed blood glucose levels in C57Bl6/J mice (■) and 129P2/OlaHsd mice (□). A:** Body weight is significantly higher in pregnant mice of both backgrounds.* ***B: *Blood glucose values are significantly higher in the mice on a C57Bl6/J background. Data are represented as mean ± SEM. *p<0.05, **p<0.01, ***p<0.001.*

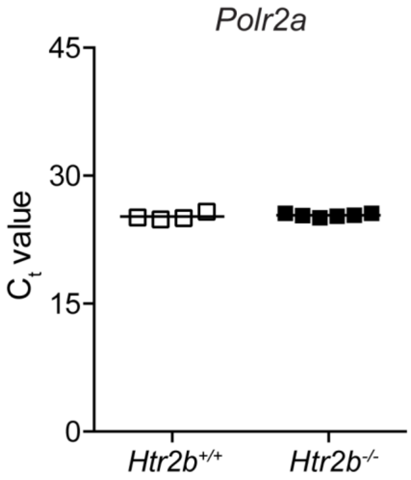

Figure S4 qRT-PCR Ct values of Polr2a in pancreatic islets of Htr2b$^{+/+}$ (□) and Htr2b$^{-/-}$ (■) mice. *Each square represents N=1. Black line represents mean.*

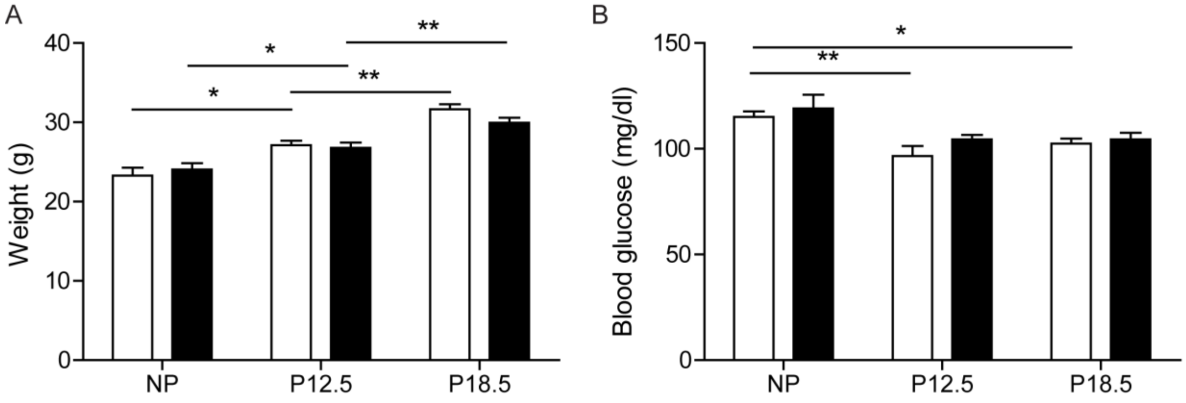

Figure S5 Body weight and random fed blood glucose levels in Htr2b$^{+/+}$ (□) and Htr2b$^{-/-}$ (■) mice do not significantly differ between groups. A: *Body weight is significantly higher at P12.5 and P18.5 compared to NP mice.* ***B:*** *In Htr2b+/+ mice, blood glucose levels are significantly lower in the pregnant mice compared to NP mice. Data are presented as mean ± SEM. *p<0.05, **p<0.01, **p<0.001.*
